# Sequential Acquisition of Human Papillomavirus Infection at Genital and Anal Sites, Liuzhou, China

**DOI:** 10.3201/eid2610.191646

**Published:** 2020-10

**Authors:** Feixue Wei, Yingying Su, Xuelian Cui, Xiaojuan Yu, Yafei Li, Qiaoqiao Song, Kai Yin, Shoujie Huang, Mingqiang Li, Jun Zhang, Ting Wu, Ningshao Xia

**Affiliations:** Xiamen University School of Public Health, Xiamen, China (F. Wei, Y. Su, X. Yu, Y. Li, Q. Song, S. Huang, J. Zhang, T. Wu, N. Xia);; Liuzhou Center for Disease Control and Prevention, Liuzhou, China (X. Cui, K. Yin, M. Li)

**Keywords:** human papillomavirus, HPV, viruses, genital sites, anal sites, sequential infection, infection, autoinoculation, sexually transmitted infections, observational cohort study, China

## Abstract

Little is known about the risk for acquiring a concordant human papillomavirus (HPV) infection in a genital (or anal) site after an anal (or genital) HPV infection. We collected 3 sets of anogenital specimens at 6-month intervals from 2,309 men and 2,378 women in Liuzhou, China, and tested these specimens for HPV. The risk for sequential anal HPV infection in participants with a previous genital HPV infection was higher than for participants without an infection (hazard ratio [HR] 4.4, 95% CI 3.4–5.8 for women and HR 2.6, 95% CI 1.4–4.6 for men). For sequential genital HPV infection, women with a previous anal infection had a higher risk (HR 1.9, 95% CI 1.2–3.1), but no major difference was found for men (HR 0.7, 95% CI 0.2–1.9). Our study indicates that autoinoculation might play a major role in anogenital HPV transmission, in addition to direct sexual intercourse, especially for anal infection in women.

Oncogenic human papillomavirus (HPV) infection can cause cancers at the anogenital site (*1*–*3*). Globally, HPV-attributable anogenital cancers include ≈570,000 cervical, 8,500 vulvar, 12,000 vaginal, 13,000 penile, and 35,000 anal cases (*4*,*5*). Although HPV spreads mainly through sexual contact, a study conducted among men who have sex with women (MSW) estimated an anal HPV infection prevalence of 12.2% (*6*). Another cohort study in Hawaii, USA, observed that women with no receptive anal sex still had anal HPV infections (*7*). These facts imply that other modes of transmission, independent of penile–anal penetration, are possible for acquiring anal HPV infections. Recently, Lin et al. (*8*) conducted a collaborative pooled analysis in paired cervical and anal samples and found a strong association between the presence of high-risk HPV (HR-HPV) at these 2 sites at the type-specific level, suggesting having the same source of infection either from the same sexual partner, autoinoculation within different anogenital sites, or both.

Two studies have assessed the risk for sequential HPV infection with a concordant genotype of an anatomic site, followed by infection at another site, and showed that autoinoculation might be a way to transmit HPV infection. One study focused on women in Hawaii and observed that the hazard ratios (HRs) for cervical-to-anal HPV infection was 20.5 (95% CI 16.3–25.7) and the HR for anal-to-cervical HPV infection was 8.33 (95% CI 6.36–12.20) (*9*). The other study focused on MSW in the United States, Brazil, and Mexico (HIM study) and reported that the HR of infection with any of the 9-valent vaccine–related types from the genital-to-anal site was 2.80 (95% CI 1.32–5.99) (*10*). However, both studies were conducted in relatively sexually active persons (e.g., 45.2% of women in Hawaii had >7 lifetime sexual partners [*7*] and 42.4% of MSW in the HIM study had >9 lifetime sexual partners [*10*)]). Whether the autoinoculation risk between genital and anal sites is similarly high in persons with relatively conservative sexual attitudes remains unknown. Furthermore, the contribution of autoinoculation for HPV infection at different anogenital sites of different sexes remains to be deeply explored.

In this study, we enrolled men and women from the general population in Liuzhou, China, of which 56% of the participants had only 1 lifetime sexual partner (*11*). The puropose of this study was to assess the risk for sequential type-specific and grouped HPV infection of genital and anal sites.

## Methods

### Study Population

During May 2014–July 2016, we conducted an observational cohort study to evaluate the natural history of genital and anal HPV infections among the general population in Liuzhou, China (*12*). Men and women who were 18–55 years of age, sexually active before enrollment, had never had an HPV vaccination, and had no plan to relocate in the next year were recruited by posters, flyers, and television advertisements. Women who were pregnant were excluded from the study (*13*). Written informed consent was obtained from each participant, and the protocol was approved by the Ethics Committee of the Liuzhou Center for Disease Control and Prevention.

### Genital and Anal HPV DNA Samples

At the enrollment visit, each participant was individually interviewed by a trained interviewer by using a questionnaire to collect baseline information on characteristics and hygienic and sexual behaviors. For women, 2 iCleanhcy-flocked swabs (Huachenyang Corporation, https://www.hcymedical.com) were independently used to collect exfoliated vaginal and vulvar samples. For men, a combined specimen from the penis, glans penis, coronary sulcus, and prepuce (if available) was collected by using a prewetted swab. For both sexes, a prewetted swab was used to sweep 360° around the perianal area and was then inserted »1.5–2.0 cm into the anal canal and rotated 360° to obtain exfoliated cells (*11*,*14*). Anogenital samples were obtained by using the same methods twice more, 6 and 12 months after enrollment.

The HPV DNA of each specimen was extracted and amplified by using the GP5+/6+ primer system. HPV genotyping was performed by using MeltPro (Zeesan Biotech Co., http://www.zeesandx.com) to test 16 different types, including 13 HR-HPV types (HPV-16, -18, -31, -33, -35, -39, -45, -51, -52, -56, -58, -59, and -68) and 3 low-risk types (HPV-6, -11, and -66). For all samples, we also tested for glyceraldehyde-3-phosphate dehydrogenase to assess the adequacy of the samples. Only glyceraldehyde-3-phosphate dehydrogenase–positive or HPV DNA–positive samples were deemed effective samples. We combined the results of vaginal and vulvar samples to represent the status of female genital HPV infection, and positive results for either vaginal or vulvar samples were defined as genital positive.

### Statistical Analysis

**Figure 1:**
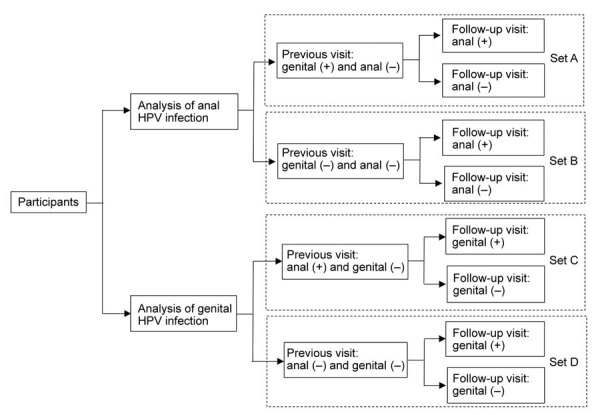
Study sets for assessment of risk for sequential genital and anal HPV infection after infection of the other site among men and women, Liuzhou, China. HPV, human papillomavirus.

We selected participants in the following 4 sets to analyze the risk for sequential genital or anal HPV infections in persons with or without a previous same-type HPV infection at the other site (Figure 1): 1) set A, genital HPV positive and anal negative at the previous visit and having >1 effective follow-up visit for the anal site; 2) set B, both genital and anal HPV negative at the previous visit and having >1 effective follow-up visit for the anal site; 3) set C, anal HPV positive and genital HPV negative at the previous visit and having >1 effective follow-up visit for the genital site; 4) set D, both anal and genital HPV negative at the previous visit and having >1 effective follow-up visit for the genital site. For each type-specific and grouped HPV type, different participants were included in the 4 sets. Persons who were in any of these sets were finally included in the analytic set of this study. Baseline characteristics of men and women included and not included in the analytic set were compared by using χ^2^ tests.

Incidence rates and 95% CIs for sequential anal HPV infection were assessed by analyzing data from set A and set B. We then calculated HRs and 95% CIs for HPV types by comparing the incidence rates between those with previous genital infections (set A) and those without previous genital infections (set B) by using Cox regression models. Similarly, we also examined the risk for sequential HPV infection in the genital site among participants with or without previous anal infection by using data for set C and set D. Incidence rates and HRs of grouped HPV (HPV-6/11; HPV-16/18; 9V-HPV: 9-valent vaccine-related HPV types, -6, -11, -16, -18, -31, -33, -45, -52, and -58; HR-HPV; and any HPV) infection were also assessed. The analysis unit for grouped infection was based on the individual, but we also performed a sensitivity analysis based on the infection. Considering that some participants might experience >1 infection during the study, a robust sandwich estimator, Wei-Lin-Weissfeld Cox regression, that adjusted for within-subject correlations, was used to analyze the HRs of grouped HPV infection. HRs and 95% CIs of sequential infection were also evaluated after excluding the influence of factors of demographics, health behavior, and sexual behavior.

The Kaplan-Meier method was used to calculate the cumulative probability of any HPV infection at the genital (or anal) site with or without a previous infection of the same genotype at the anal (or genital) site by sex. Comparison of the differences in the cumulative incidence between groups was performed by using the log-rank test. All analyses were performed by using SAS version 9.4 (SAS Institute, https://www.sas.com), and a p value <0.05 was considered statistically significant.

## Results

### Characteristics of Cohorts

Among 2,309 men and 2,378 women enrolled in the observational cohort, 1,489 (64.5%) men and 2,022 (85.0%) women who supplied effective genital and anal samples at the previous visit and having >1 effective anal or genital sample at follow-up visits were included in the analytic set (Appendix Table 1). These men were followed-up for a median of 12.5 (range 5.2–19.0) months; the women were followed-up for a median of 12.4 (range 5.1–20.1) months. The median age of the men was 40 (interquartile range 31–47) years, and the median age of the women was 39 (interquartile range 30–47) years. Compared with men, women had lower education levels, more conservative sexual behavior, and more hygienic practices. The remaining 820 men and 356 women, who were younger, more sexually active, and more likely to have unfixed sexual partners, were not included in the analytic set (Appendix Table 1).

### Incidence Rates of Sequential Anal HPV Infections among Participants with or without Previous Genital Infection by Sex

Incidence rates of anal infection were 8.8 (95% CI 5.2–14.8)/1,000 person-months for any HPV in men with previous genital infection and 3.8 (95% CI 2.9–4.9)/1,000 person-months for men without previous genital infection; the HR was 2.6 (95% CI 1.4–4.6) for previously genital-positive men versus genital-negative men (Table 1). For women, the risk for acquiring an anal infection of any HPV type was also higher in women with a preceding concordant genital HPV infection than in women without a preceding genital infection (HR 4.4, 95% CI 3.4–5.8). Similarly, increased incidence rates of sequential genital-to-anal infections were also reported for most of the other grouped HPV types in both sexes. After excluding the influence of demographics, health behaviors, and sexual behavior factors, persons with genital HPV infections at previous visits still had a higher risk for acquiring an anal infection in the follow-up visit; HRs ranged from 2.4 to 2.7 for men and from 4.1 to 4.5 for women (Appendix Table 2).

**Table 1:** Incidence rate of sequential HPV infection in the anus for participants with or without previous genital HPV infection of the same type by sex in Liuzhou, China, 2014–2016*

HPV status at genital site before anal infection	Positive for HPV type at anal site						
	Men				Women		
	No.	Incidence/1,000 person-months (95% CI)	HR (95% CI)		No.	Incidence/1,000 person-months (95% CI)	HR (95% CI)
Any HPV							
Negative	52	3.8 (2.9–4.9)	1.0		149	6.2 (5.3–7.3)	1.0
Positive	14	8.8 (5.2–14.8)	2.6 (1.4–4.6)		87	25.9 (21.0–31.9)	4.4 (3.4–5.8)
High-risk HPV							
Negative	33	2.4 (1.7–3.3)	1.0		136	5.6 (4.8–6.7)	1.0
Positive	13	8.9 (5.2–15.4)	4.2 (2.2–8.0)		82	25.2 (20.3–31.3)	4.7 (3.6–6.2)
9V-HPV							
Negative	35	2.5 (1.8–3.5)	1.0		69	2.8 (2.2–3.6)	1.0
Positive	5	6.0 (2.5–14.3)	2.8 (1.1–6.9)		32	19.8 (14.0–28.0)	7.6 (5.0–11.5)
HPV 16/18							
Negative	7	0.5 (0.2–1.1)	1.0		20	0.8 (0.5–1.3)	1.0
Positive	1	3.7 (0.5–26.0)	10.4 (1.3–85.9)		11	16.9 (9.4–30.5)	25.4 (12.0–53.6)
HPV 6/11							
Negative	20	1.4 (0.9–2.2)	1.0		13	0.5 (0.3–0.9)	1.0
Positive	1	5.4 (0.8–38.7)	4.0 (0.5–30.5)		3	15.6 (5.0–48.3)	35.4 (9.9–126.9)
HPV 6							
Negative	10	0.7 (0.4–1.3)	1.0		8	0.3 (0.2–0.7)	1.0
Positive	1	9.6 (1.4–68.2)	11.9 (1.5–96.2)		2	27.5 (6.9–110.1)	95.2 (19.7–459.9)
HPV 11							
Negative	11	0.8 (0.4–1.4)	NE		5	0.2 (0.1–0.5)	1.0
Positive	0	0	NE		1	7.8 (1.1–55.3)	43.7 (5.1–375.1)
HPV 16							
Negative	2	0.1 (0.0–0.6)	1.0		11	0.5 (0.3–0.8)	1.0
Positive	1	6.7 (0.9–47.5)	52.4 (4.7–584.6)		8	16.9 (8.5–33.9)	45.8 (17.9–117.1)
HPV 18							
Negative	5	0.4 (0.2–0.9)	NE		9	0.4 (0.2–0.7)	1.0
Positive	0	0	NE		3	13.5 (4.4–42.0)	38.0 (10.3–140.6)
HPV 31							
Negative	0	0	NE		2	0.1 (0.0–0.3)	1.0
Positive	0	0	NE		3	22.1 (7.1–68.7)	289.6 (48.2–1,740.9)
HPV 33							
Negative	4	0.3 (0.1–0.8)	1.0		12	0.5 (0.3–0.9)	1.0
Positive	1	12.4 (1.7–88.2)	71.9 (7.4–700.0)		5	30.0 (12.5–72.1)	68.3 (23.9–194.9)
HPV 35							
Negative	1	0.1 (0.0–0.5)	1.0		3	0.1 (0.0–0.4)	1.0
Positive	1	19.0 (2.7–134.5)	279.0 (17.2–4,524.7)		2	13.1 (3.3–52.4)	148.5 (20.8–1,058.9)
HPV 39							
Negative	2	0.1 (0.0–0.6)	1.0		17	0.7 (0.4–1.2)	1.0
Positive	2	9.7 (2.4–38.8)	57.4 (8.0–408.9)		15	34.6 (20.9–57.4)	46.0 (22.6–93.9)
HPV 45							
Negative	1	0.1 (0.0–0.5)	1.0		1	0.0 (0.0–0.3)	NE
Positive	1	36.5 (5.1–258.8)	381.9 (23.9–6,105.8)		0	0	NE
HPV 51							
Negative	2	0.1 (0.0–0.6)	1.0		15	0.6 (0.4–1.0)	1.0
Positive	1	11.8 (1.7–83.6)	84.0 (7.6–9,277.9)		4	21.8 (8.2–58.1)	40.9 (13.4–124.8)
HPV 52							
Negative	8	0.6 (0.3–1.2)	1.0		41	1.8 (1.3–2.5)	1.0
Positive	3	9.2 (3.0–28.4)	15.9 (4.2–60.1)		29	25.5 (17.7–36.7)	14.7 (9.1–23.7)
HPV 56							
Negative	2	0.1 (0.0–0.6)	1.0		6	0.2 (0.1–0.6)	1.0
Positive	1	8.7 (1.2–61.9)	127.6 (8.0–2,039.7)		4	18.9 (7.1–50.3)	160.2 (39.4–650.7)
HPV 58							
Negative	5	0.4 (0.2–0.9)	1.0		24	1.0 (0.7–1.5)	1.0
Positive	1	2.9 (0.4–20.9)	8.6 (0.99–74.7)		11	20.4 (11.3–36.9)	20.7 (10.1–42.3)
HPV 59							
Negative	3	0.2 (0.1–0.7)	1.0		10	0.4 (0.2–0.8)	1.0
Positive	1	10.0 (1.4–71.3)	48.5 (5.0–467.3)		8	30.4 (15.2–60.8)	69.8 (27.0–180.7)
HPV 66							
Negative	0	0	NE		5	0.2 (0.1–0.5)	1.0
Positive	0	0	NE		2	18.2 (4.5–72.7)	83.3 (16.1–430.0)
HPV 68							
Negative	0	0	NE		0	0	NE
Positive	0	NE	NE		0	NE	NE

*HRs and 95% CIs of sequential grouped HPV infection were calculated based on individual results. 9V-HPV, 9-valent vaccine-related HPV types (6, 11, 16, 18, 31, 33, 45, 52, and 58); HR-HPV, high-risk HPV types (16, 18, 31, 33, 35, 39, 45, 51, 52, 56, 58, 59, and 68); HPV, human papillomavirus; HR, hazard ratio; NE, not estimable..

**Figure 2:**
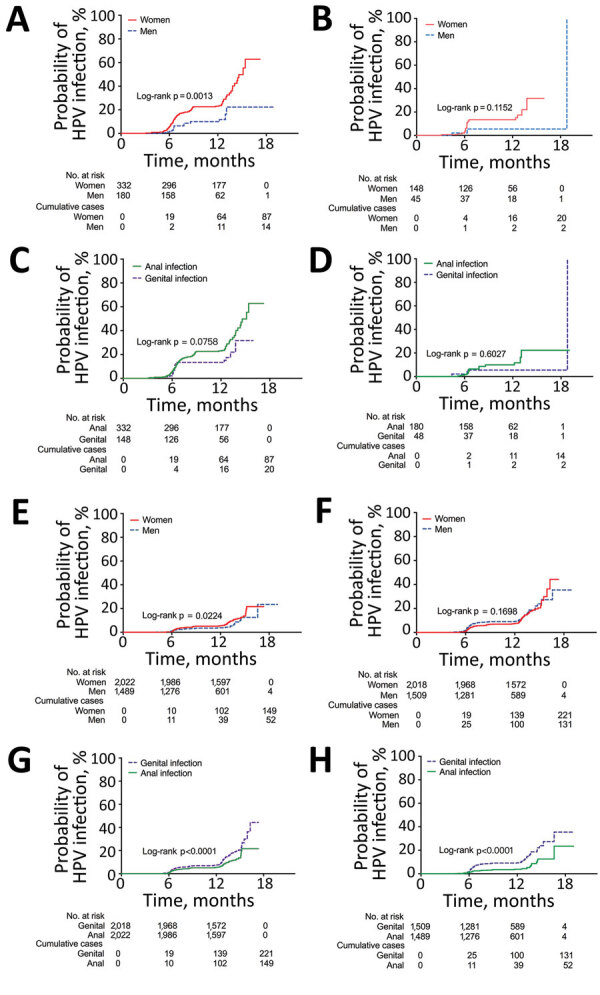
Kaplan-Meier estimates of the cumulative probability of sequential anogenital HPV infections, by sex and by site, Liuzhou, China. A) Anal HPV infection in participants with previous genital infection, by sex; B) genital HPV infection in participants with previous anal infection, by sex; C) genital or anal HPV infection in women with previous anal or genital infection, by site; D) genital or anal HPV infection in men with previous anal or genital infection, by site; E) anal HPV infection in participants without previous genital infection, by sex; F) genital HPV infection in participants without previous anal infection, by sex; G) genital or anal HPV infection in women without previous anal or genital infection, by site; H) genital or anal HPV infection in men without previous anal or genital infection, by site. HPV, human papillomavirus.

When the analytic unit was based on infection, the risks of sequential genital-to-anal grouped HPV infection in both sexes were still statistically significant (all p<0.05) and had higher HRs (Appendix Table 3). For example, for any HPV infection, the HR of genital-to-anal HPV infection was 33.6 (95% CI 18.5–61.0) for men and 55.8 (95% CI 42.9–72.7) for women. In the type-specific HPV analysis, patients of both sexes who had previous genital HPV infection had higher incidence rates of subsequent anal HPV infection. Compared with men, women with previous genital infection had a higher risk of acquiring an anal infection (p = 0.0013) (Figure 2, panel A). A sex difference was also found in anal incidence among participants without previous genital infection for any HPV type (p = 0.0224) (Figure 2, panel E).

### Incidence Rates of Sequential Genital HPV Infections among Participants with or without Previous Anal Infection by Sex

The risk for sequential genital infection with any HPV in women who had an anal HPV infection at previous visits was 1.9 times (95% CI 1.2–3.1) higher than that for women who had no anal HPV infection (Table 2). The HRs of anal-to-genital HPV infection remained significantly different when we excluded the effect of demographics, health behaviors, and sexual behaviors (all p<0.05) (Appendix Table 4).

**Table 2:** Incidence rate of sequential HPV infection at the genital site for participants with or without previous anal HPV infection of the same type by sex in Liuzhou, China, 2014–2016*

HPV status at anal site before genital infection	Positive for HPV type at genital site						
	Men				Women		
	No.	Incidence/1,000 person-months (95% CI)	HR (95% CI)		No.	Incidence/1,000 person-months (95% CI)	HR (95% CI)
Any HPV							
Negative	131	9.5 (8.0–11.2)	1.0		221	9.3 (8.1–10.6)	1.0
Positive	3	6.9 (2.2–21.4)	0.7 (0.2–1.9)		20	15.0 (9.7–23.3)	1.9 (1.2–3.1)
High-risk HPV							
Negative	116	8.4 (7.0–10.0)	1.0		201	8.4 (7.3–9.7)	1.0
Positive	2	5.8 (1.4–23.0)	0.6 (0.2–1.9)		17	13.5 (8.4–21.7)	1.9 (1.1–3.1)
9V-HPV							
Negative	67	4.8 (3.8–6.1)	1.0		98	4.1 (3.3–4.9)	1.0
Positive	3	9.3 (3.0–28.9)	1.5 (0.5–4.6)		5	7.3 (3.0–17.6)	2.1 (0.8–5.2)
HPV 16/18							
Negative	30	2.1 (1.5–3.0)	1.0		37	1.5 (1.1–2.1)	NE
Positive	1	28.6 (4.0–203.2)	21.6 (2.4–197.9)		0	0	NE
HPV 6/11							
Negative	16	1.1 (0.7–1.9)	1.0		23	0.9 (0.6–1.4)	1.0
Positive	1	10.7 (1.5–75.7)	12.3 (1.5–98.7)		2	21.4 (5.3–85.4)	34.4 (7.6–155.2)
HPV 6							
Negative	13	0.9 (0.5–1.6)	NE		13	0.5 (0.3–0.9)	1.0
Positive	0	0	NE		2	24.5 (6.1–97.8)	65.3 (14.1–302.2)
HPV 11							
Negative	3	0.2 (0.1–0.7)	1.0		10	0.4 (0.2–0.8)	NE
Positive	1	14.7 (2.1–104.7)	100.7 (9.1–1,117.4)		0	0	NE
HPV 16							
Negative	18	1.3 (0.8–2.0)	1.0		25	1.1 (0.7–1.6)	NE
Positive	1	44.5 (6.3–316.0)	51.1 (6.7–387.8)		0	0	NE
HPV 18							
Negative	12	0.9 (0.5–1.5)	NE		13	0.5 (0.3–0.9)	NE
Positive	0	0	NE		0	0	NE
HPV 31							
Negative	5	0.4 (0.1–0.9)	1.0		5	0.2 (0.1–0.5)	NE
Positive	1	31.6 (4.4–224.2)	7.8 (0.1–556.0)		0	0	NE
HPV 33							
Negative	3	0.2 (0.1–0.7)	NE		9	0.4 (0.2–0.7)	1.0
Positive	0	0	NE		1	20.3 (2.9–144.1)	109.0 (12.7–937.1)
HPV 35							
Negative	3	0.2 (0.1–0.7)	NE		7	0.3 (0.1–0.6)	NE
Positive	0	0	NE		0	0	NE
HPV 39							
Negative	21	1.5 (1.0–2.3)	NE		24	1.0 (0.7–1.5)	1.0
Positive	0	0	NE		2	15.7 (3.9–62.7)	17.5 (4.1–74.7)
HPV 45							
Negative	4	0.3 (0.1–0.8)	NE		4	0.2 (0.1–0.4)	NE
Positive	0	0	NE		0	0	NE
HPV 51							
Negative	6	0.4 (0.2–1.0)	NE		28	1.2 (0.8–1.7)	1.0
Positive	0	0	NE		2	15.4 (3.9–61.6)	12.3 (2.7–55.0)
HPV 52							
Negative	28	2.0 (1.4–3.0)	NE		50	2.2 (1.7–3.0)	1.0
Positive	0	0	NE		9	22.6 (11.8–43.5)	13.9 (6.8–28.5)
HPV 56							
Negative	4	0.3 (0.1–0.8)	NE		18	0.7 (0.5–1.2)	1.0
Positive	0	0	NE		1	14.2 (2.0–100.5)	20.5 (2.7–155.3)
HPV 58							
Negative	12	0.9 (0.5–1.6)	NE		27	1.2 (0.8–1.7)	1.0
Positive	0	0	NE		2	7.3 (1.8–29.1)	6.0 (1.4–25.3)
HPV 59							
Negative	14	1.0 (0.6–1.7)	NE		22	0.9 (0.6–1.4)	NE
Positive	0	0	NE		0	0	NE
HPV 66							
Negative	2	0.1 (0.0–0.6)	NE		5	0.2 (0.1–0.5)	1.0
Positive	0	0	NE		1	46.2 (6.5–327.6)	339.2 (30.2–3,815.6)
HPV 68							
Negative	0	0	NE		1	0.0 (0.0–0.3)	NE
Positive	0	0	NE		0	0	NE

*HRs and 95% CIs of sequential grouped HPV infection were calculated based on individual results. 9V-HPV, 9-valent vaccine-related HPV types (6, 11, 16, 18, 31, 33, 45, 52, and 58); high-risk HPV types (16, 18, 31, 33, 35, 39, 45, 51, 52, 56, 58, 59, and 68); HPV, human papillomavirus; HR, hazard ratio; NE, not estimable.

In addition, the risk for genital HR-HPV, 9V-HPV, and HPV 6/11 infections in women also increased after anal HPV infection with a concordant type. For men, sex differences were only found in anal-to-genital sequential infections of types 16/18 and 6/11. However, the results based on the infection analysis showed that the risk for acquiring all grouped HPV infections in a genital site was higher in both sexes among participants with previous infections in the anal site versus those without previous infections (Appendix Table 5). For each HPV type analysis, previous anal HPV infection was strongly associated with the sequential genital concordance type of HPV infection, particularly for types 11, 16, and 31 in men and types 6, 33, 39, 51, 52, 56, 58, and 66 in women (Table 2). We found no sex difference in the cumulative probability of sequential genital infections either in previously infected or uninfected persons (Figure 2, panels B, F).

### Differences in Sequential Genital and Anal Infections among Participants with or without Previous HPV Infection at Anal or Genital Sites

Among participants without previous HPV infection at the other site, incidence rates for genital HPV infection were higher than those for anal HPV infection in men and women (both p<0.0001) (Figure 2, panels G, H). However, for participants with previous HPV infection at the other site, participants were more likely to acquire an anal infection than a genital infection, although the difference was not significant (p = 0.0758 for women and p = 0.6027 for men) (Figure 2, panels C, D).

## Discussion

In both sexes, we observed a dramatically increased risk for acquiring sequential HPV infection at the anal site among participants who were genitally positive for concordant HPV types in previous visits compared with persons who had genitally negative results. In a similar fashion, an increased risk for sequential acquisition of a genital HPV infection was also observed in both sexes with a previous concordant anal HPV infection compared with those without previous anal infection, although no significant difference was found for any HPV and HR-HPV types in men on the basis of individual calculation. However, for type-specific HPV, men with a previous anal infection had a higher risk for acquiring a genital infection. Furthermore, if we calculated HRs on the basis of infection instead of person, we found that men with previous anal HPV infection had higher risk for sequentially acquiring genital infection for any HPV types than those without previous anal infection (HR 8.8, 95% CI 3.1–24.8). For genital-to-anal and anal-to-genital infections, women had higher risk than men, which might be caused by differences between female mucosal epithelium and male keratinized epithelium and the sex differences in the anatomy of genitalia.

In both sexes, if no previous HPV infection was present in the other site, the incidence of HPV infection was higher at the genital site than at the anal site. It is easy to interpret this phenomenon as reflecting the finding that genital intercourse is always the major sexual behavior in women and heterosexual men (*15*–*18*). However, if previous HPV infection existed at the other site, the incidence of concordant HPV infection increased dramatically in the anus and became even higher than that at the genital site, although a significant difference was not reached. These data implied that, for anal HPV infection, genital-to-anal transmission might play a greater role than direct intercourse for women and also make a relatively major contribution for men. This conclusion is concordant with a previous study in which a history of anal sex was not significantly associated with sequential acquisition of an incident anal infection after a concordant HPV infection of the cervix (*9*).

In the same cohort in Liuzhou, we observed a longer persistence of any HPV type in both genital and anal sites in women compared with men (*12*,*19*). This observation is concordant with the data in the analysis we describe in this article, showing that the HRs of incident infection by any type of HPV seems higher in women who were positive for the same HPV type at the other site versus women who were negative compared with men. In addition, HR-HPV infection at the genital site was more difficult to clear than the anal infection in both sexes (*12*), which could partially explain the relatively high HRs in genital-to-anal infection compared with anal-to-genital infection in men and women.

Similar phenomena were observed in the only 2 previous studies that focused on sequential HPV acquisition between anal and genital sites (*9*,*10*). Goodman et al. reported a high risk for cervical-to-anal HPV infection and anal-to-cervical HPV infection with respective HRs of 14.2 (95% CI 9.86–20.5) and 7.08 (95% CI 3.94–12.7) for HR-HPV types in women in Hawaii (*9*). The other study focused on sequential HPV infection among anogenital sites in MSW from the HIM study and found that the HR of genital-to-anal infection with 9V-HPV was 2.61 (95% CI 1.20–6.00) and of anal-to-genital infection with 9V-HPV was 1.18 (95% CI 0.64–2.01) (*10*).

Given that 99.7% of participants in our study were heterosexual, along with the results of the 2 studies just described (*9*,*10*), we can well understand the observations that heterosexual men and women not engaging in anal sex also have positive HPV samples at the anal site (*6*,*7*,*13*). These infections might be acquired by other methods, such as autoinoculation and partner-assisted inoculation (including use of a sex toy or digital sex). This suggestion was reported in previous studies, which showed that digital sex (performed by self or sexual partners) can cause transmission events between the genital site and anal site in both sexes (*20*,*21*).

A nonsexual habit might also result in autoinoculation. Simpson et al. (*22*) studied the associations among different wiping habits after urination/defecation and anal HPV infection or related conditions. Results showed that front-to-back wiping after urination or defecation were both related to an increased prevalence of anal HR-HPV, abnormal anal cytology, and histologically proven neoplasia. More studies should be conducted to clarify the association between types of nonsexual behaviors and anal–genital HPV infection.

The strengths of our study are the large sample size and the use of the same methods to collect and test anogenital samples from males and females, which make it possible to directly compare sex differences in sequential infection among different sites. Despite these strengths, there are still limitations. Information regarding anal sex among participants was not collected, which made it impossible to analyze the relationship between sexual behavior at anal site and anal HPV infection. As is the case for other studies focused on sequential analysis of HPV infection at different sites (*9*,*10*), there is no way to know the previous HPV status of the transmitted site before baseline visit. Therefore, it is possible that the sequential HPV infection was a latent infection rather than a transmission event from the transmission site.

In conclusion, men and women with previous HPV infection at the genital or anal site had a higher risk for sequentially acquiring a concordant HPV infection at the other site. For anogenital HPV infection, autoinoculation of HPV might play a major role, in addition to that of sexual intercourse, especially for anal HPV infection in women. Therefore, there is no need to focus on anal sexual intercourse and its associated stigma when discussing anal cancer and its prevention.

Appendix Additional information on sequential acquisition of human papillomavirus infection at genital and anal sites, Liuzhou, China.
