# Supplementary material for: Sequential Acquisition of Human Papillomavirus Infection at Genital and Anal Sites, Liuzhou, China
Source: Emerg Infect Dis. 2020 Oct;26(10):2387–93. doi: 10.3201/eid2610.191646 (PMC7510729; doi:10.3201/eid2610.191646)
Supplement: Appendix — Additional information on sequential acquisition of human papillomavirus infection at genital and anal sites, Liuzhou, China. [file 19-1646-Techapp-s1.pdf]

Article DOI: https://doi.org/10.3201/eid2610.191646

Sequential Acquisition of Human Papillomavirus Infection at Genital and Anal Sites, Liuzhou, China

Appendix

Appendix Table 1. Baseline demographic characteristics of men and women who were in and not in the analytic set for papillomavirus infection at genital and anal sites, Liuzhou, China, 2014–2016*

	In analytic set†		Not in analytic set‡	
Factor	Men, n = 1,489	Women, n = 2,022	Men, n = 820	Women, n = 356
Age, y§¶				
18–25	207 (13.9)	302 (15.0)	217 (26.5)	157 (44.1)
26–35	363 (24.4)	546 (27.0)	261 (31.8)	94 (26.4)
36–45	461 (31.0)	569 (28.1)	200 (24.4)	52 (14.6)
46–55	458 (30.8)	605 (29.9)	142 (17.3)	53 (14.9)
Median (IQR)	40 (31–47)	39 (30–47)	33 (25–42)	27 (22–38)
Mean (SD)	38.5 (9.9)	38.0 (10.0)	33.8 (10.2)	30.6 (10.4)
Residence				
Rural	594 (39.9)	853 (42.2)	300 (36.6)	134 (37.6)
Urban	895 (60.1)	1,169 (57.8)	520 (63.4)	222 (62.4)
Ethnicity§¶#				
Han	615 (41.3)	787 (38.9)	368 (44.9)	131 (36.8)
Miao	283 (19.0)	554 (27.4)	177 (21.6)	69 (19.4)
Zhuang	305 (20.5)	450 (22.3)	164 (20.0)	106 (29.8)
Others	286 (19.2)	231 (11.4)	111 (13.5)	50 (14.0)
Education, y#				
<12	1,192 (80.0)	1,672 (82.7)	645 (78.7)	284 (79.8)
≥12	297 (20.0)	350 (17.3)	175 (21.3)	72 (20.2)
Annual household income, CNY#				
<50,000	1,128 (75.8)	1,611 (79.7)	605 (73.8)	279 (78.4)
≥50,000	361 (24.2)	411 (20.3)	215 (26.2)	77 (21.6)
Marital status§¶#				
Single/divorced/separated/widowed	311 (20.9)	281 (13.9)	266 (32.4)	130 (36.5)
Married/cohabitating	1,178 (79.1)	1,741 (86.1)	554 (67.6)	226 (63.5)

Factor	In analytic set†		Not in analytic set‡	
	Men, n = 1,489	Women, n = 2,022	Men, n = 820	Women, n = 356
Had had a sauna#				
No	1,007 (67.6)	1,492 (73.8)	550 (68.2)	255 (71.6)
Yes	482 (32.4)	530 (26.2)	261 (31.8)	101 (28.4)
Used a towel supplied by a sauna#				
No	1,080 (72.5)	1,633 (80.8)	596 (72.7)	273 (76.7)
Yes	409 (27.5)	389 (19.2)	224 (27.3)	83 (23.3)
Stayed in a hotel§¶#				
No	282 (18.9)	703 (34.8)	109 (13.3)	104 (29.2)
Yes	1,207 (81.1)	1,319 (65.2)	711 (86.7)	252 (70.8)
Used a towel supplied by a hotel§¶#				
No	588 (39.5)	1,294 (64.0)	283 (34.5)	178 (50.0)
Yes	901 (60.5)	728 (36.0)	537 (65.5)	178 (50.0)
Age at time of first sexual intercourse, y§¶#				
<18	94 (6.3)	106 (5.2)	96 (11.7)	39 (11.0)
18–25	1,150 (77.2)	1,699 (84.0)	619 (75.5)	300 (84.3)
>25	245 (16.5)	217 (10.7)	105 (12.8)	17 (4.8)
Lifetime no. sexual partners§¶#				
1	711 (47.8)	1,360 (67.3)	329 (40.1)	194 (54.5)
2–3	459 (30.8)	580 (28.7)	272 (33.2)	146 (41.0)
≥4	319 (21.4)	82 (4.1)	219 (26.7)	16 (4.5)
No. sexual partners within past year§¶#				
0–1	1,291 (86.7)	1,940 (95.9)	671 (81.8)	330 (92.7)
>1	198 (13.3)	82 (4.1)	149 (18.2)	26 (7.3)
Sexual orientation				
Homosexual	1,486 (99.8)	2,016 (99.7)	816 (99.5)	355 (99.7)
Heterosexual	3 (0.2)	6 (0.3)	4 (0.5)	1 (0.3)
Sexual partners ever having sex with others				
No	561 (37.7)	812 (40.2)	313 (38.2)	159 (44.7)
Yes	61 (4.1)	92 (4.5)	43 (5.2)	16 (4.5)
Unknown	867 (58.2)	1,118 (55.3)	464 (56.6)	181 (50.8)
Previous STD diagnosis¶#				
No	1,371 (92.1)	1,671 (82.6)	766 (93.4)	313 (87.9)
Yes	118 (7.9)	351 (17.4)	54 (6.6)	43 (12.1)
Frequency of condom use¶#				
Not always	1,369 (91.9)	1,923 (95.1)	738 (90.0)	326 (91.6)
Always	120 (8.1)	99 (4.9)	82 (10.0)	30 (8.4)

Factor	In analytic set†		Not in analytic set‡	
	Men, n = 1,489	Women, n = 2,022	Men, n = 820	Women, n = 356

Values are no. (%) unless indicated otherwise. CNY, Chinese Yuan; HPV, human papillomavirus; IQR, interquartile range.

†Participants in analytic set included those who supplied effective genital and anal samples at previous visit and effective anal or genital samples at ≥1 follow-up visit.

‡Participants not in analytic set included those who participated in the study, whereas their samples were invalid for the previous visit, or they provided valid sample for the previous visit, whereas no valid sample was provided at the follow-up visit.

§Significant difference between men included in and not included in the analysis set at p<0.05 by χ^2 test or Fisher exact test.

¶Significant difference between women included in and not included in the analysis set at p<0.05 by χ^2 test or Fisher exact test.

#Significant difference between men and women included in the analysis set at p<0.05 by χ^2 test or Fisher exact test.

Appendix Table 2. Association between previous genital HPV infection with sequential anal HPV infection excluding effect of other demographics, health behaviors, and sexual behaviors, by sex, Liuzhou, China, 2014–2016*

Factor	Men		Women	
	HR (95% CI)	p value	HR (95% CI)	p value
Total	2.6 (1.4–4.6)	0.0019	4.4 (3.4–5.8)	<0.0001
Age	2.6 (1.4–4.7)	0.0023	4.2 (3.3–5.5)	<0.0001
Residence	2.7 (1.5–4.9)	0.0010	4.5 (3.4–5.8)	<0.0001
Ethnicity	2.7 (1.5–4.9)	0.0010	4.4 (3.4–5.7)	<0.0001
Education	2.6 (1.4–4.7)	0.0016	4.4 (3.4–5.7)	<0.0001
Annual household income	2.5 (1.4–4.6)	0.0022	4.4 (3.4–5.7)	<0.0001
Marital status	2.6 (1.4–4.7)	0.0018	4.2 (3.3–5.5)	<0.0001
Had a sauna	2.5 (1.3–4.5)	0.0034	4.4 (3.4–5.7)	<0.0001
Used a towel supplied by a sauna	2.4 (1.3–4.5)	0.0036	4.4 (3.4–5.7)	<0.0001
Stayed in a hotel	2.5 (1.4–4.6)	0.0019	4.4 (3.4–5.7)	<0.0001
Used a towel supplied by a hotel	2.5 (1.4–4.6)	0.0018	4.3 (3.3–5.6)	<0.0001
Age at time of first sexual intercourse	2.6 (1.4–4.7)	0.0016	4.3 (3.3–5.6)	<0.0001
Lifetime no. sexual partners	2.6 (1.4–4.7)	0.0016	4.1 (3.1–5.4)	<0.0001
No. sexual partners within past year	2.5 (1.4–4.6)	0.0020	4.2 (3.3–5.5)	<0.0001
Orientation	2.6 (1.5–4.8)	0.0014	4.4 (3.4–5.8)	<0.0001
Sexual partners ever having sex with others	2.5 (1.4–4.5)	0.0024	4.4 (3.4–5.8)	<0.0001
Previous STD diagnosis	2.6 (1.4–4.6)	0.0019	4.4 (3.4–5.7)	<0.0001
Frequency of condom use	2.6 (1.4–4.7)	0.0017	4.4 (3.4–5.8)	<0.0001

*HPV, human papillomavirus; HR, hazard ratio; STD, sexually transmitted disease.

Appendix Table 3. Incidence rate of grouped sequential HPV infection in anal site among participants with or without previous genital HPV infection of the same type, by sex, Liuzhou, China, 2014–2016*

	Positive for HPV type at anal site					
	Men			Women		
HPV status at genital site	No. events	Incidence rate/1,000 person-months (95% CI)	HR (95% CI)	No. events	Incidence rate/1,000 person-months (95% CI)	HR (95% CI)
Any HPV						
Negative	56	0.3 (0.2–0.3)	1.0	169	0.4 (0.4–0.5)	1.0
Positive	14	7.7 (4.5–12.9)	33.6 (18.5–61.0)	97	22.7 (18.6–27.6)	55.8 (42.9–72.7)
High-risk HPV						
Negative	35	0.2 (0.1–0.3)	1.0	151	0.5 (0.4–0.6)	1.0
Positive	13	8.0 (4.6–13.7)	45.5 (23.8–87.2)	92	23.2 (18.9–28.4)	51.5 (39.3–67.6)
9V-HPV						
Negative	46	0.4 (0.3–0.5)	1.0	113	0.5 (0.4–0.6)	1.0
Positive	8	6.3 (3.2–12.7)	19.8 (9.4–41.9)	62	21.2 (16.5–27.2)	43.7 (32.1–59.6)
HPV 16/18						
Negative	7	0.3 (0.1–0.5)	1.0	20	0.4 (0.3–0.6)	1.0
Positive	1	3.6 (0.5–25.3)	19.8 (2.4–163.3)	11	15.9 (8.8–28.6)	46.9 (22.1–99.4)
HPV 6/11						
Negative	21	0.8 (0.5–1.2)	1.0	13	0.3 (0.2–0.5)	1.0
Positive	1	5.3 (0.7–37.5)	7.5 (1.0–56.7)	3	14.9 (4.8–46.3)	67.4 (18.7–242.5)

*HRs and 95% CIs of sequential grouped HPV infection were calculated on the basis of infection. HPV, human papillomavirus; HR, hazard ratio.

Appendix Table 4. Association between previous anal HPV infection with sequential genital HPV infection excluding the effect of other demographics, health behaviors, and sexual behaviors, by sex, Liuzhou, China, 2014–2016*

	Men		Women	
Factor	HR (95% CI)	p value	HR (95% CI)	p value
Total	0.7 (0.2–1.9)	0.4603	1.9 (1.2–3.1)	0.0078
Age	0.7 (0.2–2.0)	0.4285	1.7 (1.1–2.8)	0.0282
Residence	0.7 (0.2–1.9)	0.4604	1.8 (1.1–3.0)	0.0141
Ethnicity	0.7 (0.2–2.0)	0.4903	1.8 (1.1–2.9)	0.0173
Education	0.7 (0.2–1.9)	0.4551	1.9 (1.2–3.1)	0.0079
Annual household income	0.6 (0.2–1.8)	0.618	1.9 (1.2–3.0)	0.0101
Marital status	0.7 (0.2–1.9)	0.4568	1.7 (1.0–2.7)	0.0390
Had a sauna	0.7 (0.2–2.0)	0.4983	1.8 (1.1–2.9)	0.0146
Used a towel supplied by a sauna	0.7 (0.2–1.9)	0.4806	1.8 (1.1–2.9)	0.0185

	Men		Women	
Factor	HR (95% CI)	p value	HR (95% CI)	p value
Stayed in a hotel	0.7 (0.2–1.9)	0.4640	1.8 (1.1–3.0)	0.0124
Used a towel supplied by a hotel	0.7 (0.2–1.9)	0.4556	1.8 (1.1–2.9)	0.0208
Age at time of first sexual intercourse	0.7 (0.2–1.9)	0.4528	1.9 (1.2–3.0)	0.0109
Lifetime no. sexual partners	0.7 (0.3–2.0)	0.5317	1.7 (1.1–2.7)	0.0293
No. of sexual partners within the past year	0.6 (0.2–1.7)	0.3405	1.8 (1.1– 2.9)	0.0201
Orientation	0.7 (0.2–1.9)	0.4585	1.9 (1.2–3.1)	0.0077
Sexual partners ever having sex with others	0.7 (0.2–1.9)	0.4458	1.9 (1.2–3.1)	0.0077
Previous STD diagnosis	0.7 (0.2–1.9)	–0.4518	1.9 (1.2–3.1)	0.0072
Frequency of condom use	0.7 (0.2–1.9)	0.4609	1.9 (1.2–3.1)	0.0069

*HPV, human papillomavirus; HR, hazard ratio; STD, sexually transmitted disease.

Appendix Table 5. Incidence rate of grouped sequential HPV infections at genital sites among participants with or without previous anal HPV infection of the same type, by sex, in Liuzhou, China, 2014–2016*

	Positive for HPV type at genital site					
HPV status at anal site before genital infection	Men			Women		
	No events	Incidence rate/,1000 person-months (95% CI)	HR (95% CI)	No. events	Incidence rate/1,000 person-months (95% CI)	HR (95% CI)
Any HPV						
Negative	148	0.7 (0.6–0.8)	1.0	261	0.7 (0.6–0.8)	1.0
Positive	3	6.0 (1.9–18.6)	8.8 (3.1–24.8)	20	13.0 (8.4–20.1)	23.0 (14.4–36.7)
High-risk HPV						
Negative	130	0.7 (0.6–0.9)	1.0	233	0.8 (0.7–0.9)	1.0
Positive	2	5.2 (1.3–20.9)	6.4 (1.9–21.5)	17	12.0 (7.5–19.3)	18.9 (11.3–31.5)
9V-HPV						
Negative	98	0.8 (0.6–1.0)	1.0	156	0.7 (0.6–0.9)	1.0
Positive	3	7.3 (2.4–22.7)	8.5 (3.0–24.3)	14	12.2 (7.2–20.6)	21.1 (12.3–36.2)
HPV 16/18						
Negative	30	1.1 (0.8–1.5)	1.0	38	0.8 (0.6–1.1)	NE
Positive	1	28.6 (4.0–203.2)	42.8 (4.7–392.3)	0	0	NE
HPV 6/11						
Negative	16	0.6 (0.4–0.9)	1.0	23	0.5 (0.3–0.7)	1.0
Positive	1	8.9 (1.3–63.3)	20.0 (2.8–142.0)	2	18.7 (4.7–74.9)	53.8 (15.6–185.8)

*HRs and 95% CIs for sequential grouped HPV infections were calculated on the basis of infection. HPV, human papillomavirus; HR, hazard ratio; NE, not estimated.
